# The median effective concentration of propofol in combination with different doses of esketamine during gastrointestinal endoscopy in adults

**DOI:** 10.3389/fphar.2022.1034236

**Published:** 2022-10-20

**Authors:** Miaomiao Feng, Gaoxiang Shi, Wenjing Cui, Ning Zhang, Qipeng Xie, Weiwei Zhang

**Affiliations:** ^1^ Shanxi Bethune Hospital, Shanxi Academy of Medical Sciences, Tongji Shanxi Hospital, Third Hospital of Shanxi Medical University, Taiyuan, China; ^2^ Tongji Hospital, Tongji Medical College, Huazhong University of Science and Technology, Wuhan, China

**Keywords:** esketamine, propofol, gastroscopy, colonoscopy, median effective concentration

## Abstract

We designed a four-arm randomized controlled trial to investigate the median effective concentration (EC_50_) of propofol in combination with different doses of esketamine inducing appropriate depth of anaesthesia during gastrointestinal endoscopy in adults. One hundred patients aged 18–65 years planning for gastrointestinal endoscopy were divided into four groups randomly: esketamine 0, 0.15, 0.25 and 0.5 mg/kg groups (*n* = 25). Propofol doses followed the Dixon and Massey up-and-down method with different starting between groups. The primary endpoint was the EC_50_ of propofol. Secondary outcomes included the cumulative dose of propofol, the duration of the procedure, recovery time, and adverse effects. The EC_50_ (median, 95% confidence interval) of propofol was significantly less in the esketamine 0.5 mg/kg group compared with the esketamine 0, 0.15, and 0.25 mg/kg groups [1.34 (1.15, 1.54) vs. 3.48 (3.25, 3.71), 2.82 (2.58, 3.07), and 2.36 (2.11, 2.61), respectively; *p* < 0.001]. The total dose of propofol (mean ± SD) required for the whole procedure was significantly less in the esketamine 0.5 mg/kg group compared with the esketamine 0, 0.15, and 0.25 mg/kg groups [95.5 ± 43.1 vs. 277.4 ± 49.0, 207.8 ± 31.6, and 135.1 ± 27.7, respectively; *p* < 0.001]. The recovery time was significantly longer in esketamine 0 and 0.5 mg/kg group compared with other two groups (*p* < 0.001). More patients in the esketamine 0.5 mg/kg group experienced visual disturbance compared with the other groups (*p* = 0.016). Additionally, the incidence of hypotensionin the esketamine 0 mg/kg group after inducation was higher compared with other groups (*p* < 0.001). In summary, the administration of esketamine significantly and dose-dependently reduced the dose of propofol required to accomplish procedures.

## Introduction

Gastroscopy and colonoscopy are important and common endoscopic methods for the diagnosis and treatment of gastrointestinal and colorectal diseases, such as the screening of early gastric cancer. They are invasive procedures and may cause stress reactions in the body, such as nausea, retching, vomiting, abdominal pain, abdominal distension, tachycardia and hypertension (Zheng et al., 2018). Painless endoscopy can effectively relieve the pain and discomfort of patients, and has become the preferred clinical diagnosis and treatment measure. Because the process is typically brief, the need for anaesthetic might have a quick onset and wake-up time, improving the work effect.

Despite widespread use and expertise, there is no standardized drug protocol for this condition (Li et al., 2019). How to improve the effect of sedation and analgesia, to choose a safe and reliable anesthesia regimen has been the focus and difficulty of clinic. Propofol is widely used in procedural sedation/anaesthesia due to its rapid onset, rapid metabolism and minimal side effects (Shinsuke et al., 2014). However, propofol may result in hypotension, bradycardia, and respiratory depression. Researches (Zhou et al., 2016; Li et al., 2019; Thompson et al., 2019) have shown that the use of propofol combined with an adjuvant, such as ketamine or an opioid (sufentanil), for procedural sedation/anaesthesia is more efficacious and safe compared with either an opioid/benzodiazepine combination or propofol alone. Ketamine has irreplaceable advantages in maintaining spontaneous breathing and sympathomimetic properties, the combination of ketamine and propofol is preferred over the combination of opioids and propofol, for the latter may increase the possibility of respiratorydepression.

Esketamine is an N-methyl-D-aspartate receptorantagonist, has similar pharmacological effects to ketamine, such asanaesthetic, analgesic and sympathomimetic properties (SmithApeldoorn et al., 2020; Bonaventura et al., 2021). Studies (Wang et al., 2019; Eberl et al., 2020) have shown the advantages using esketamine combined with propofol. However, no study has reported the optimal esketamine-propofol proportion during endoscopic procedures.

Therefore, we conducted a double-blind randomised controlled trial to determine the median effective concentration (EC_50_) of propofol incombination with different doses of esketamine and explore the type and frequency of adverse events associated with the propofol-esketamine dose combinations.

## Materials and methods

### Study design and patients

This single-centre, prospective, randomised, controlled, double-blinded trial was performed from January 2022 to August 2022 at Shanxi Bethune Hospital, Shanxi, China. The protocol was approved by the local ethics committee (Medical Ethics Committee of Shanxi Bethune Hospital) and registered at Chinese Clinical Trial Registry (http://www.chictr.org, ChiCTR2100044567). After informed written consent of patient, we included 100 subjects aged 18–65 years, American Society of Anesthesiologists (ASA) class I or II, BMI 18.5–27.9 kg/m^2^, undergoing painless gastrointestinal endoscopy. Exclusion criteria included: 1) allergy to any of the drugs used in the study; 2) preoperative use of analgesic and sedative drugs, or have a history of heavy smoking, alcohol abuse, serious drug abuse, and severe systemic infections; 3) renal or hepatic dysfunction; 4) a history of unregulated or malignant hypertension, significant heart disease, elevated intracranial pressure and intraocular pressure, psychiatric disease, pregnancy or suckling period.

### Randomisation and masking

Subjects were allocated sequentially using random number generating computer software in a 1:1:1:1 ratio to receive one of four doses of esketamine: 0 (control group), 0.15, 0.25, or 0.5 mg/kg. Randomisation envelopes containing the study medication data were prepared. The envelopes were opened by the single study investigator (C.P.) preparing the study medication immediately before the patient arrived in the operating theatre. The investigator collecting data, patients and their guardians, endoscopists and nurses were blinded to the study medication. The allocation sequence was not available to any member of the research team until databases had been completed and locked.

### Procedures

All patients were fasted at least 8 h before the gastrointestinal endoscopy. After intravenous access was obtained, an infusion of 250 ml NaCl 0.9% was started. Pulse oximetry, electrocardiography and blood pressure were routinely monitored, and patients were asked to position the mselvesin lateral decubitus position, and oxygen was given for 4 L/min through a nasal catheter. The study investigator (C.P.), who did not participate in anesthesia during the endoscopy, prepared the study medications. The dose of esketamine was drawn into a 10 ml syringe and saline was added to produce a 10 ml solution. Patients were sedated using a TCI of propofol (Diprivan1%; Corden Pharma S.P.A, Caponago, Italy). We used the Graseby 3500 (AstraZeneca, United Kingdom) infusion pump preprogrammed for propofol TCI in the Marsh model.

Lidocaine 1%, 0.05 mg/kgwas administered i.v. to minimisepain from propofol injection (Parmar and Koay, 1998). Approximately 10 s after lidocaine administration, propofol was injected. After the patient’s eyelash reflexes disappear, esketamine was administered according to the study group assignment. Propofol TCI was initiated before administration of esketamine to avoid possible psychogenic effects of esketamine (Eberl et al., 2020). Two minutes after the administration, endoscope insertion was attempted and the patient’s response was recorded. If the patient was apneic, endoscope insertion was delayed until spontaneous ventilation resumed. If the patient was still too responsive to tolerate the procedure, additional doses of propofol 0.5–1 mg/kg was provided. After examination patients were transferred to the recovery room, and they had to stay in the recovery room for at least 30 min. Patients were discharged home once awake and with vital signs in the normal range, able to ambulate without assistance, and without significant side effects such as nausea.

### Determination of EC_50_

We used an “up-and-down” titration method as described to determination of EC_50_ of propofol (Dixon, 1991; Pace and Stylianou, 2007). For each group, the starting propofol dose of the first patient was estimated to be close to the anticipated EC_50_ (Table 1) (Hayes et al., 2018). A positive response was defined as coughing, gagging, or excessive movement, that prevented complete insertion of the endoscope. If the response of the preceding patient was positive, the starting propofol dose in the subsequent patient in each group was increased by 0.5 μg/ml. The starting propofol dose in the subsequent patient in each group was decreased by 0.5 μg/ml if the previous patient tolerated complete insertion of the endoscope.

**TABLE 1:** Drug dosage of esketamine and propofol for each group.

Group	Esketamine dose (mg/kg)	Propofol starting dose (μg/ml)	Doseinterval (μg/ml)
E_0_	0	3	0.5
E_1_	0.15	2.5	0.5
E_2_	0.25	2	0.5
E_3_	0.5	1.5	0.5

### Secondary end-point parameters

The heart rate (HR), and pulse oxygen saturation (SpO_2_) were monitored constantly until discharge, and the non-invasive arterial blood pressure (SBP/DBP/MAP) was measured at three minutesintervals throughout the procedure. All vital signs were recorded at the following six time points: before induction of anesthesia (T0; baseline), at the end of anaesthesia induction (T1), at the time of endoscope insertion (T2), at the end of endoscopy (T3), when patients arrived at PACU (T4) and when patients awoke (T5). Patients with severe hypotension (systolic blood pressure [SBP] of <80 mmHg) or bradycardia (HR of <45 bpm) during the procedure who required rescue cardiovascular drugs were excluded from the study, the same effect-site concentration was used for the substitute patient.

The cumulative dose of propofol during the procedure, and the duration of the procedure (time of scope insertion to time of scope removal), Recovery time is defined as the time when propofol is stopped until the patient is awake without associated complications.

During the procedure, the following adverse events were recorded: the occurrence of hypotension, the occurrence of respiratory depression (SpO_2_ < 90%, lasting for at least 10 s), need for airway management: such as jaw thrust, insertion of oropharyngeal or nasopharyngeal airway, or positive pressure ventilation. During the recovery room, the following adverse events were recorded: the occurrence of nausea/vomiting, dizziness, visual disturbance, such as blurred or double-vision, nystagmus, and hallucinations.

### Statistical analysis

The primary end point was to determine the EC_50_ of propofol with esketamine at 0, 0.15, 0.25 and 0.5 mg/kg for gastrointestinal endoscopy in adults. The second outcomes were total dose of propofol, recovery time, perioperative adverse events and so on. The EC_50_ of propofol calculated as the average of midpoints of failure-success crossovers, a probit analysis was used to estimate the EC_95_ value.

Numerical data were analyzed using a one-way ANOVA, Bonferroni method was used for comparison tests between groups, and categorical data were analyzed with the χ^2^ test. The alterations of the MAP and HR were evaluated by a repeated measure analysis of variance (ANOVA). Continuous data are reported as mean (SD), or median and interquartile range, as appropriate. Categorical data are reported as frequencies (%). A value of *p* < 0.05 were considered statistically significant. The analysis was performed using SPSS for Windows (version 22.0; SPSS, Inc., Chicago, IL, United States).

The sample size was calculated according to the stop rule of the up-and-down sequential methodology, in each group, at least six cross overs are needed for accurate EC_50_ calculation, and anesthesia trials using Dixon’s up and down method typically have 20–40 patients (Pace and Stylianou, 2007; Feng et al., 2020; Oron et al., 2022; Zheng et al., 2022).

## Results

**FIGURE 1:**
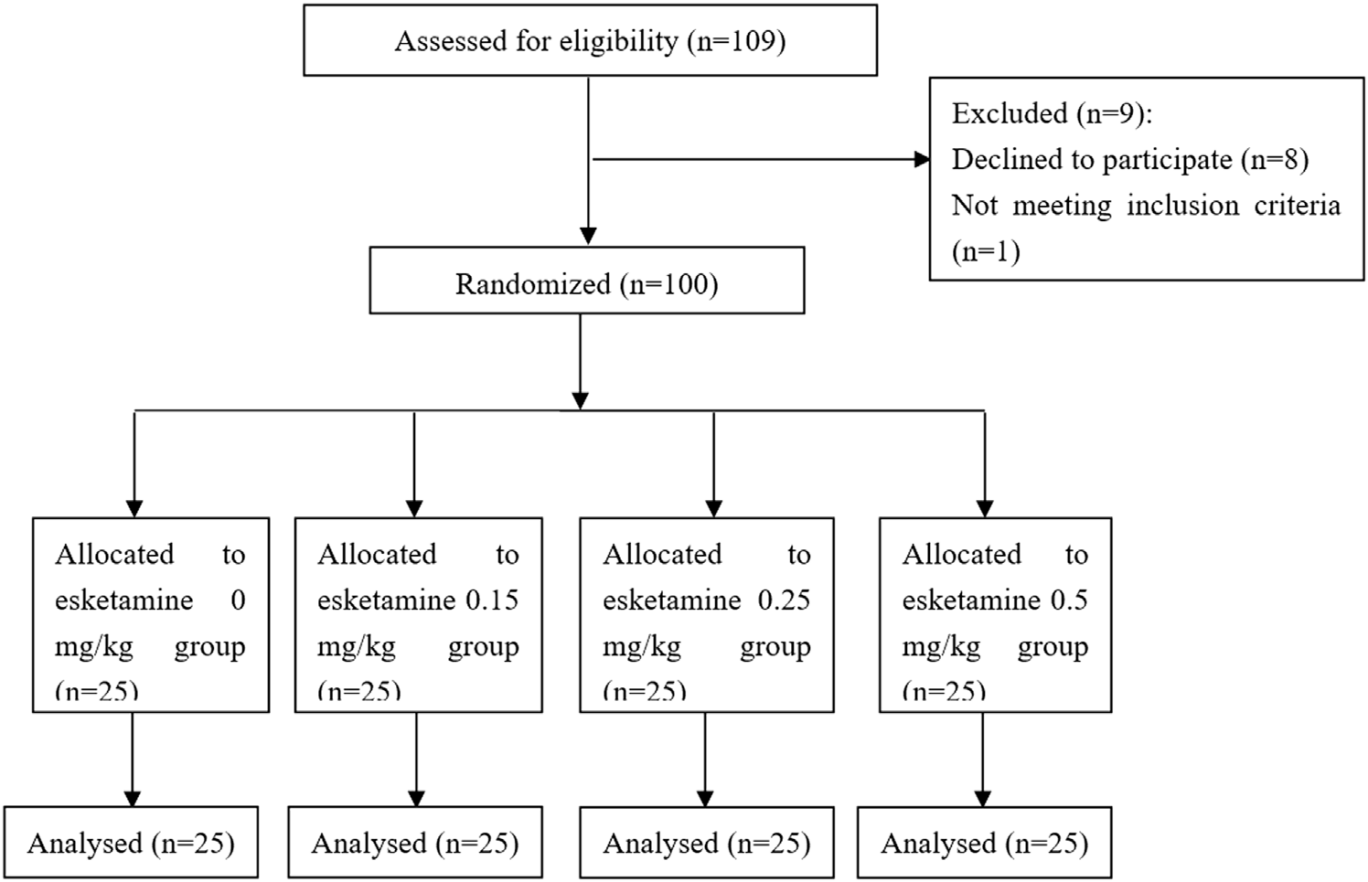
Flow diagram of included participants.

**FIGURE 2:**
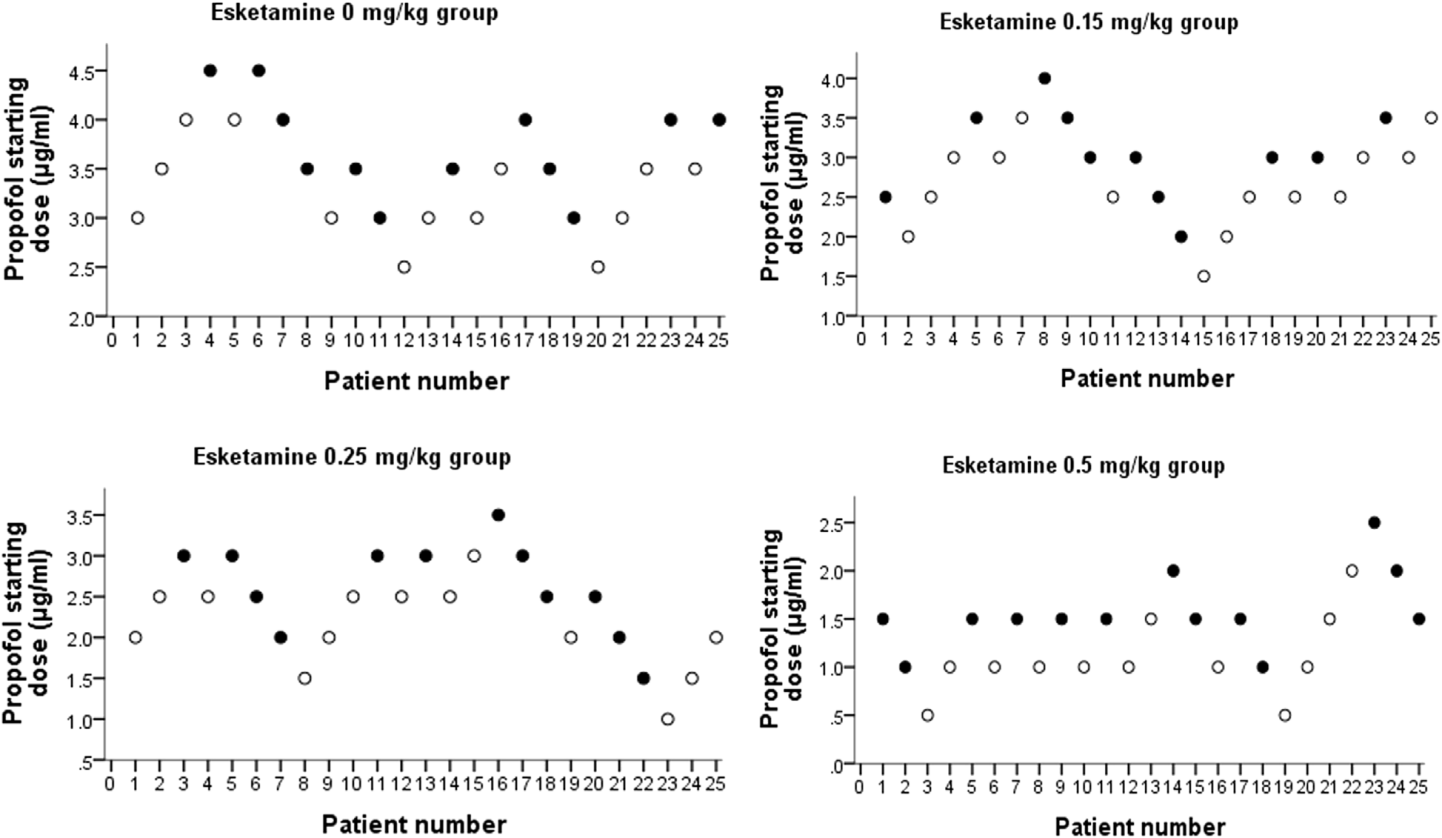
Dixon up-and-down plots for four groups. “●” represents failure, and “○” represents success.

109 patients were approached; eight declined to participate and one participant was excluded because ofnot meeting inclusion criteria (Figure 1). There were no significant differences in demographic data or procedural factors among subjects in the four groups (Table 2). The Dixon up-and-down sequences for each group are presented in Figure 2. The EC_50_ (95% CI) of propofol was significantly lower in the esketamine 0.5 mg/kg group compared with the esketamine 0, 0.15, and 0.25 mg/kg groups [1.34 (1.15, 1.54) vs. 3.48 (3.25, 3.71), 2.82 (2.58, 3.07), and 2.36 (2.11, 2.61), respectively; *p* < 0.001] (Table 3). There was no statistically significant difference between the ketamine 0.15 and 0.25 mg/kg groups. The total dose of propofol (mean ± SD) required for the whole procedure was significantly less in the esketamine 0.5 mg/kg group compared with the esketamine 0, 0.15, and 0.25 mg/kg groups [95.5 ± 43.1 vs. 277.4 ± 49.0, 207.8 ± 31.6, and 135.1 ± 27.7, respectively; *p* < 0.001]. The recovery time was significantly longer in the esketamine 0 and 0.5 mg/kg groups compared with other two groups (*p* < 0.001; Table 2).

**TABLE 2:** Patient characteristics.

	Esketamine group (dose mg/kg)					
	0 (*n* = 25)	0.15 (*n* = 25)	0.25 (*n* = 25)	0.5 (*n* = 25)	*p* value	F/Chi-square value
Gender (M/F)	14/11	12/13	15/10	11/14	0.659	1.603
Age (yr)	52.6 ± 6.5	54.4 ± 8.7	53.9 ± 6.9	50.7 ± 9.3	0.357	1.091
Height (cm)	166.2 ± 7.4	164.8 ± 7.2	164.2 ± 5.9	163.8 ± 7.8	0.641	0.562
Weight (kg)	62.8 ± 7.9	62.0 ± 6.8	61.6 ± 6.8	62.9 ± 9.8	0.927	0.155
BMI (kg/m2)	22.7 ± 2.2	22.8 ± 2.0	22.8 ± 1.7	23.3 ± 2.3	0.712	0.458
ASA (I/II)	17/8	16/9	17/8	19/6	0.828	0.888
Duration of procedure (min)	16.3 ± 2.3	16.4 ± 2.5	16.1 ± 2.6	16.9 ± 4.0	0.808	0.325
Total dosage of propofol (mg)	277.4 ± 49.0	207.8 ± 31.6	135.1 ± 27.7	95.5 ± 43.1	<0.001*	107.411
Awakening time (min)	10.9 ± 2.6	7.4 ± 2.6	7.4 ± 2.8	12.7 ± 3.1	<0.001*	22.377

Note: Data are presented as mean ± SD or number, as appropriate. *A value of *p* < 0.001 was considered significant.

**TABLE 3:** EC_50_ and EC_95_ with 95% confidence intervals of propofol in four groups, based on the Dixon–Massey Up-and-Down Sequential Allocation Method and Probit Regression respectively. EC_50_, effective concentration in 50% of patients. EC_95_, effective concentration in 95% of patients. *p <0.001 was considered significantly different between groups.

	E0 (esketamine 0 mg/kg)	E1 (esketamine 0.15 mg/kg)	E2 (esketamine 0.25 mg/kg)	E3 (esketamine 0.5 mg/kg)	*p* value
Dixon–Massey EC_50_ (μg/mL)	3.48 (3.25, 3.71)	2.82 (2.58, 3.07)	2.36 (2.11, 2.61)	1.34 (1.15, 1.54)	<0.001*
Probit regression EC_50_ (μg/mL)	3.53 (3.04, 4.04)	2.97 (2.51, 3.50)	2.41 (1.92, 2.92)	1.29 (0.79, 1.77)	<0.001*
Probit regression EC_95_ (μg/mL)	4.90 (4.32, 6.23	4.34 (3.75, 5.73)	3.78 (3.20, 5.11)	2.66 (2.10, 3.94)	<0.001*

**FIGURE 3:**
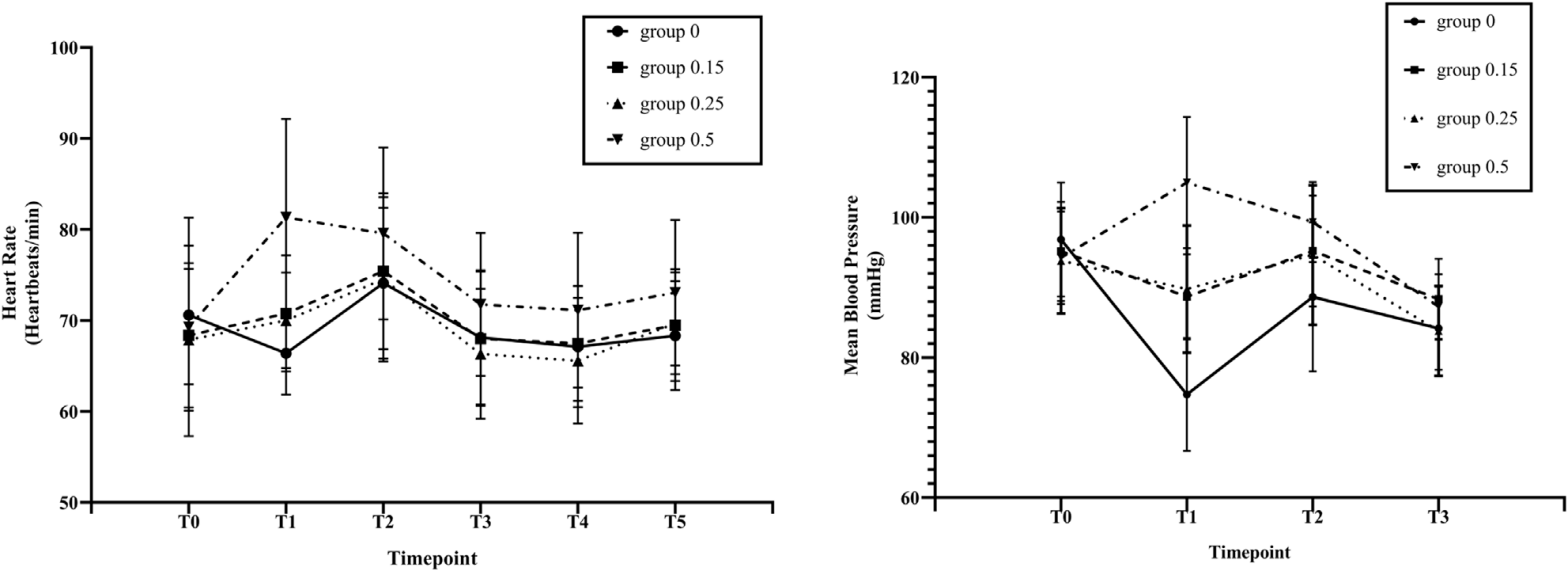
Changes in heart rate (HR) and mean blood pressure (MBP) by study groups across different study time points. Before induction of anesthesia (T0; baseline), at the end of anaesthesiainduction (T1), at the time of endoscope insertion (T2), at the end of endoscopy (T3), at arrived at PACU (T4) and at patients’ awakening (T5). Group 0, esketamine 0 mg/kg group;Group 0.15, esketamine 0.15 mg/kg group; Group 0.25, esketamine 0.25 mg/kg group; Group 0.5, esketamine 0.5 mg/kg group. Data are presented as means and vertical bars denote standard deviation.

As shown in Figure 3, in the esketamine 0.5 mg/kg group, the HR of T1 and T2 increased significantly (*p* < 0.05) compared with T0. At the end of anesthesia induction (T1), the HR in esketamine 0.5 mg/kg group was significantly higher than that in esketamine 0, 0.15, and 0.25 mg/kg groups (*p* < 0.05). There was a significant decrease in MBP in esketamine 0 and 0.15 mg/kg groups after finishing induction (T1), MBP of T1, T2 and T3 were significantly lower than that of baseline in the esketamine 0 mg/kg group (*p* < 0.05). While in the esketamine 0.5 mg/kg group, there was a significant increasein MBP after finishing induction (T1), and the MBP of T1 significant higher than those of T0, T2, and T3 of the same group (*p* < 0.05). The MAP was significantly lower in the esketamine 0 mg/kg group compared with the other three groups after finishing induction (*p* < 0 .05). In contrast, The MAP was significantly higher in the esketamine 0.5 mg/kg group than in the other three groups (*p* < 0.05).

During the procedure, there was no significant difference in the occurrence of respiratory depression, nausea/vomiting, and dizziness among groups. More patients in the esketamine 0.5 mg/kg group experienced visual disturbance compared with the other groups. In addition, the incidence of hypotension in the esketamine 0 mg/kg group after inducation was higher compared with other groups (Table 4). All the adverse events were self-limited and transient.

**TABLE 4:** Adverse events.

	Esketamine 0 mg/kg group	Esketamine 0.15 mg/kg group	Esketamine 0.25 mg/kg group	Esketamine 0.5 mg/kg group	*p* value	Chi-square value
Hypotension	13	1	1	0	<0.001*	36.0
Respiratory depression	5	3	4	1	0.377	3.095
Nausea/vomiting	2	0	0	1	0.286	3.780
Dizziness	1	2	2	3	0.780	1.087
Visual disturbance	0	4	3	8	0.016*	10.275

Note: Hypotension was defined as more than 20% decrease in MBP when compared to baseline. Respiratory depression was defined as oxygen saturation less than 90% and lasting for at least 10 seconds. Visual disturbances included blurred or double-vision, nystagmus, and hallucinations. *p < 0.05 was considered significant.

## Discussion

We have demonstrated that the EC_50_ of propofol to achieve satisfactory sedation conditions decreased as the dose of esketamine increased from 0 to 0.5 mg/kg in ASA I or II patients. Patients who received esketamine 0 mg/kg or 1 mg/kg had a high incidence of either propofol or esketamine-related adverse events.

To our knowledge, this is the first randomised controlled dose-finding study on the influence of esketamine on the EC_50_ of propofol anaesthesia during gastrointestinal endoscopy in adults. Propofol is often used in combination with ketamine and opioids to improve efficacy and patient tolerance. Several studies (Wang et al., 2019; Eberl et al., 2020; Huang et al., 2022; Xu et al., 2022) have compared a single-dose combination of esketamine and propofol with propofol and other types of drugs in terms of side effects and degree of sedation undergoing minor procedures, withesketamine doses ranged from 0.15 to 0.5 mg/kg. Esketamine (0.5 mg/kg) has been proven to be safe and well tolerated in Chinese patients undergoing painless gastroscopy, with no serious adverse events (Wang et al., 2019). While a recent study (Zhan et al., 2022) showed that 0.2 mg/kg esketamine combined with propofol is safe and effective for painless gastrointestinal endoscopy. Eberl et al. (2020) demonstrated that low-dose esketamine (0.15 mg/kg) reduces the requirement of propofol for sedation during ERCP when compared with alfentanil. Since the optimal dose ratio of propofol to esketamine is unknown, the empirical medications carry a potential risk of overdose. Therefore, our study was designed specifically to assess the optimal esketamine-propofol proportion for the procedures.

Our findings show that EC_50_ of propofol when co-administration with 0.15, 0.25 or 0.5 mg/kg esketamine was decreased by 19.0%, 32.2% and 61.5% compared with 0 mg/kg group. Consistent with our results, a significant dose-dependent reduction in the EC_50_ of propofol by esketamine has been reported in elderly patients (Yang et al., 2021). In this study, patients were divided into three groups receiving 0, 0.25, and 0.5 mg/kg of esketamine, respectively. The results showed that EC_50_ of propofol when co-administration with 0.25 or 0.5 mg/kg esketamine was decreased by 33.6% and 53.7% compared with 0 mg/kg group.

It is well known that propofol may cause dose-dependent bradycardia and hypotension, due to reduced systemic vascular resistance and myocardial contractility (Claeys et al., 1988; Lee, 2004). Our study also showed that the incidence of hypotension was significantly higher in esketamine 0 mg/kg group than in the other groups. While propofol co-administration with 0.5 mg/kg esketamine, there was a significant increasein MBP after finishing induction, which may be due to the decrease of propofol dosage and the increase of cardiac output by esketamine (Kamp et al., 2021). Similarly, Xin et al. (2020) and Tu et al. (2021) also showed that propofol combined with esketamine had better hemodynamic stability. In contrast to previous study (Yang et al., 2021), our study found that esketamine 0.15 and 0.25 mg/kg groups had shorter recovery times than esketamine 0 and 0.5 mg/kg group. The difference in the recovery time between the two studies is due to the different definition of the recovery time. Also, we found a significant increase in the incidence of visual disturbances in the esketamine 0.5 mg/kg group, a known adverse effect of esketamine (Guo et al., 2022), which may also explain the longer recovery time in this group.

There are some limitations to the study that should be mentioned. The first is the lack of esketamine dose more than 0.5 mg, such as 1 mg/kg, the choice of esketamine doses was based on the clinical experience of the investigators and previous studies. Although a higher esketamine dose can reduce propofol dose and associated side effects more significantly, it may also significantly increase the incidence of esketamine-related adverse effects. The cognitive function changes and psychomimetic side effects caused by esketamine have been widely concerned, which could be dose-related (Zheng et al., 2022). Second, in this study, EC_95_ was calculated using data extrapolation and bootstrapping, and it has a relatively large 95% confidence interval value that should be interpreted with caution. Further research can be conducted using, for example, biased coin design up-and-down sequential method (BCD-UDM). Finally, only patients with ASA physical status I-II were included in this study; therefore, the results are not applicable to clinically critical patients.

## Conclusion

In conclusion, this report suggests that increasing the dose of esketamine can significantly reduce the dose of propofol required to accomplish procedures, with a corresponding reduction in propofol associated hemodynamic changes.

## Data Availability

The original contributions presented in the study are included in the article/Supplementary Material, further inquiries can be directed to the corresponding author.
